# Designing Interoperable Health Care Services Based on Fast Healthcare Interoperability Resources: Literature Review

**DOI:** 10.2196/44842

**Published:** 2023-08-21

**Authors:** Jingwen Nan, Li-Qun Xu

**Affiliations:** 1 Health IT Research China Mobile (Chengdu) Industrial Research Institute Chengdu China

**Keywords:** Health level 7 Fast Healthcare Interoperability Resources, HL7 FHIR, interoperability, literature review, practice guideline, mobile phone

## Abstract

**Background:**

With the advent of the digital economy and the aging population, the demand for diversified health care services and innovative care delivery models has been overwhelming. This trend has accelerated the urgency to implement effective and efficient data exchange and service interoperability, which underpins coordinated care services among tiered health care institutions, improves the quality of oversight of regulators, and provides vast and comprehensive data collection to support clinical medicine and health economics research, thus improving the overall service quality and patient satisfaction. To meet this demand and facilitate the interoperability of IT systems of stakeholders, after years of preparation, Health Level 7 formally introduced, in 2014, the Fast Healthcare Interoperability Resources (FHIR) standard. It has since continued to evolve. FHIR depends on the Implementation Guide (IG) to ensure feasibility and consistency while developing an interoperable health care service. The IG defines rules with associated documentation on how FHIR resources are used to tackle a particular problem. However, a gap remains between IGs and the process of building actual services because IGs are rules without specifying concrete methods, procedures, or tools. Thus, stakeholders may feel it nontrivial to participate in the ecosystem, giving rise to the need for a more actionable practice guideline (PG) for promoting FHIR’s fast adoption.

**Objective:**

This study aimed to propose a general FHIR PG to facilitate stakeholders in the health care ecosystem to understand FHIR and quickly develop interoperable health care services.

**Methods:**

We selected a collection of FHIR-related papers about the latest studies or use cases on designing and building FHIR-based interoperable health care services and tagged each use case as belonging to 1 of the 3 dominant innovation feature groups that are also associated with practice stages, that is, data standardization, data management, and data integration. Next, we reviewed each group’s detailed process and key techniques to build respective care services and collate a complete FHIR PG. Finally, as an example, we arbitrarily selected a use case outside the scope of the reviewed papers and mapped it back to the FHIR PG to demonstrate the effectiveness and generalizability of the PG.

**Results:**

The FHIR PG includes 2 core elements: one is a practice design that defines the responsibilities of stakeholders and outlines the complete procedure from data to services, and the other is a development architecture for practice design, which lists the available tools for each practice step and provides direct and actionable recommendations.

**Conclusions:**

The FHIR PG can bridge the gap between IGs and the process of building actual services by proposing actionable methods, procedures, and tools. It assists stakeholders in identifying participants’ roles, managing the scope of responsibilities, and developing relevant modules, thus helping promote FHIR-based interoperable health care services.

## Introduction

### Background

The development and innovation of health care service models have accelerated the demand for data exchange and service interoperability. In the United States, the Health Information Technology for Economic and Clinical Health Act took effect in 2009, specifying health IT–based systems as an integrated part of the country’s health care reform. It has spurred the electronic health record (EHR) adoption rate through reward and punishment measures [1]. In addition, the US Department of Health and Human Services established a specific agency, the Office of the National Coordinator for Health Information Technology, to accelerate the implementation of advanced medical IT standards, promote the exchange of electronic health care information, and improve the quality of health care services throughout the country. In Canada, the federal government funded an independent, not-for-profit organization called Canada Health Infoway, tasked with accelerating the adoption of digital health solutions, such as EHR, across the country. The government has set a 10-year implementation strategy for EHR in cooperation with the Canadian Institute for Health Information [2]. Japan has made great efforts to develop remote health care technology and has established a communication system among regional institutions by implementing electronic medical records (EMRs) in the form of an app or software as a service [3]. In China’s state health system, major public hospitals administered by national, provincial, and local health authorities are the pioneers in reforms. Over the years, the government has issued a series of policies promoting coordinated care among health care institutions at different levels of the health system [4,5], together with many qualitative or quantitative assessment criteria that guide the establishment of high-standard EMR system, regional information interoperability, and intelligent service and management in hospitals. In summary, the demand for tiered and coordinated care delivery among health care institutions worldwide is increasing rapidly, and the requirement for health care data exchange continues unabated.

The enhancement of interoperability is required by transforming health care service models and tackling the challenges of societal problems. According to a United Nations report [6], the share of the population aged ≥65 years is expected to increase from 9.3% in 2020 to approximately 16% in 2050. The rapid aging of the population unavoidably increases the burden of chronic disease care, bringing about the requirements for people-centered and continuous care delivery built on the foundation of a robust primary health care system. Therefore, it is necessary to enhance health IT system interoperability to bridge the gap between uneven health care resource distribution, remove the barrier of isolated data islands, and comprehensively improve the quality of health care services.

### Health Level 7 Fast Healthcare Interoperability Resources

**Figure 1:**
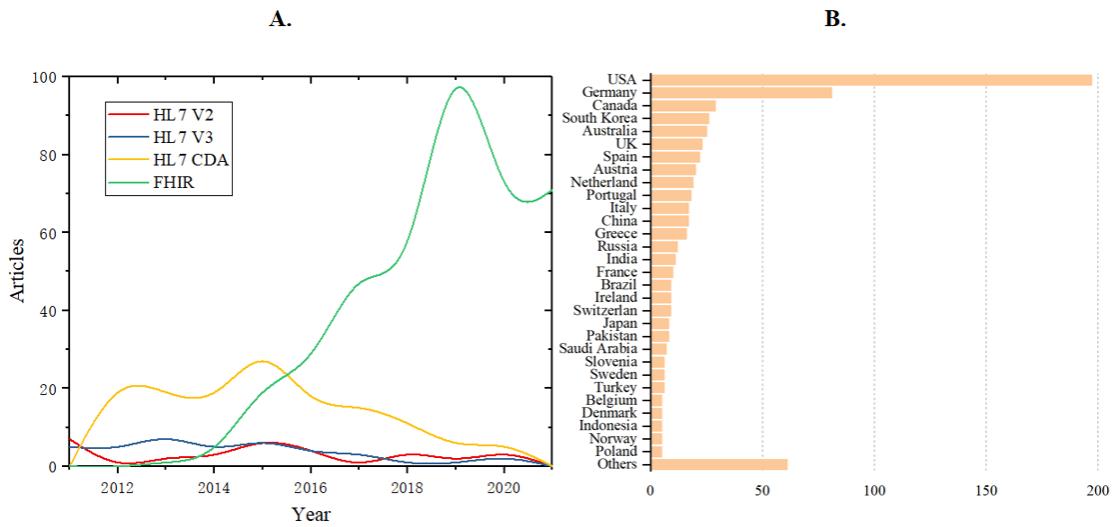
Works of literature that focus on health data standards. (A) The attention to Fast Healthcare Interoperability Resources (FHIR) has risen rapidly within a short time of its first release, far exceeding HL7 version 2, HL7 version 3, and Clinical Document Architecture (CDA) standards. (B) The United States, Germany, and Canada are the top 3 countries that published the most literature on FHIR. HL: Health Level.

Health Level 7 (HL7), founded in 1987, is a not-for-profit, standards-developing organization dedicated to providing a comprehensive framework and related standards for the exchange, integration, sharing, and retrieval of electronic health information that supports clinical practice and the management, delivery, and evaluation of health services. It has successively released many standards, including HL7 version 2, HL7 version 3, and Clinical Document Architecture (CDA). However, with the constant evolution of the internet and the thriving of the application programming interface (API) economy, digital services or assets of health organizations tend to be exposed even more widely in the form of APIs. In this context, HL7 formally introduced Fast Healthcare Interoperability Resources (FHIR) in 2014, highlighting the core concept of resources, and thus, creating a new era for health care service interoperability. A resource is the smallest exchangeable logical unit in FHIR. Resources are independent of each other but can be linked or assembled through specific rules to meet diverse service requirements. FHIR combines web standards to support resource operations through RESTful API in XML or JavaScript Object Notation format. Compared with other alternative standards, FHIR has more advantages and potential, such as comprehensive coverage of data definitions, substantial flexibility of data exchange, explicit semantics, and many available open-source tools, among others. Therefore, it has attracted constant and favorable attention from health care stakeholders since its first release, as shown in Figure 1.

We investigated the literature from the Web of Science and plotted 2 statistical charts in Figure 1. Figure 1A shows the promotion trends of different health data standards. By using the search term “HL7 v2,” “HL7 v3,” “HL7 CDA,” and “FHIR,” we identified the corresponding papers in the Web of Science database from 2010 to 2022. The results show that the attention paid to FHIR has increased rapidly within a short time, far exceeding the HL7 version 2, HL7 version 3, and CDA standards. Figure 1B compares FHIR-relevant literature among different countries. We used the search term “FHIR” to find the corresponding papers in the Web of Science database from 2014 to 2022. By reading each paper’s abstract and the corresponding author’s information, we identified the country to which the work belongs. Countries that record <5 papers fall into the “others” category. The chart shows that the United States, Germany, and Canada were the top 3 countries that published the most studies on FHIR, accounting for 28.39% (197/694), 11.67% (81/694), and 4.18% (29/694), respectively.

In addition to the dissemination activities of enthusiastic researchers and pioneering health IT ecosystem players, national health policy makers also play a pivotal role in FHIR adoption, as evidenced by the actions in the United Kingdom, United States, and Canada [7]. Overall, FHIR has gradually gained worldwide recognition and acceptance, and it has the most potential for future large-scale promotion in the health care ecosystem.

### Objectives

Owing to the growing popularity of FHIR, some academic researchers have authored review papers from their perspectives in the last few years. Ayaz et al [8] searched for FHIR-related papers published between 2012 and 2019 in 6 databases (ACM, IEEE, Springer, Google Scholar, PubMed, and ScienceDirect) and selected 80 papers for review. They found that FHIR is identical in supporting intelligent technologies, such as smartphones, tablets, mobile health apps, smartwatches, and fitness trackers, which could solve numerous health care problems that were impossible for the previous standards. Lehne et al [9] searched for FHIR-related papers in 2 databases (Web of Science and PubMed) up to 2019 and selected 131 papers for review. The statistical results revealed that data model–related topics mainly focusing on constructing profiles to implement FHIR in specific scenarios were the most attractive direction. At the same time, analytics-related topics concerning data analysis, modeling, machine learning, and more were less attractive because most FHIR projects were still in the initial development phase, dealing with implementation and data definitions rather than large-scale data analysis. Barker and Johnson[10] surveyed 734 apps released up to December 2020 in 5 digital health care application libraries (hosted by Cerner, Epic, Allscripts, Athenahealth, and Substitutable Medical Applications Reusable Technologies [SMART]) and measured their support for FHIR. They found that the number of apps that support the FHIR standard had increased from 19% in 2019 to 22% in 2020.

However, to our knowledge, there is a lack of systematic reviews that focus on the FHIR practice. A gap remains between the FHIR Implementation Guide (IG) and building actual services because IGs are rules specifying no methods, procedures, or tools. Thus, stakeholders may feel it nontrivial to participate in the ecosystem, giving rise to the need for a more actionable practice guideline (PG) for promoting FHIR’s fast adoption. Therefore, this study proposed a general FHIR PG to facilitate stakeholders in the health care ecosystem to understand FHIR and quickly develop interoperable health care services.

## Methods

### Article Selection

**Figure 2:**
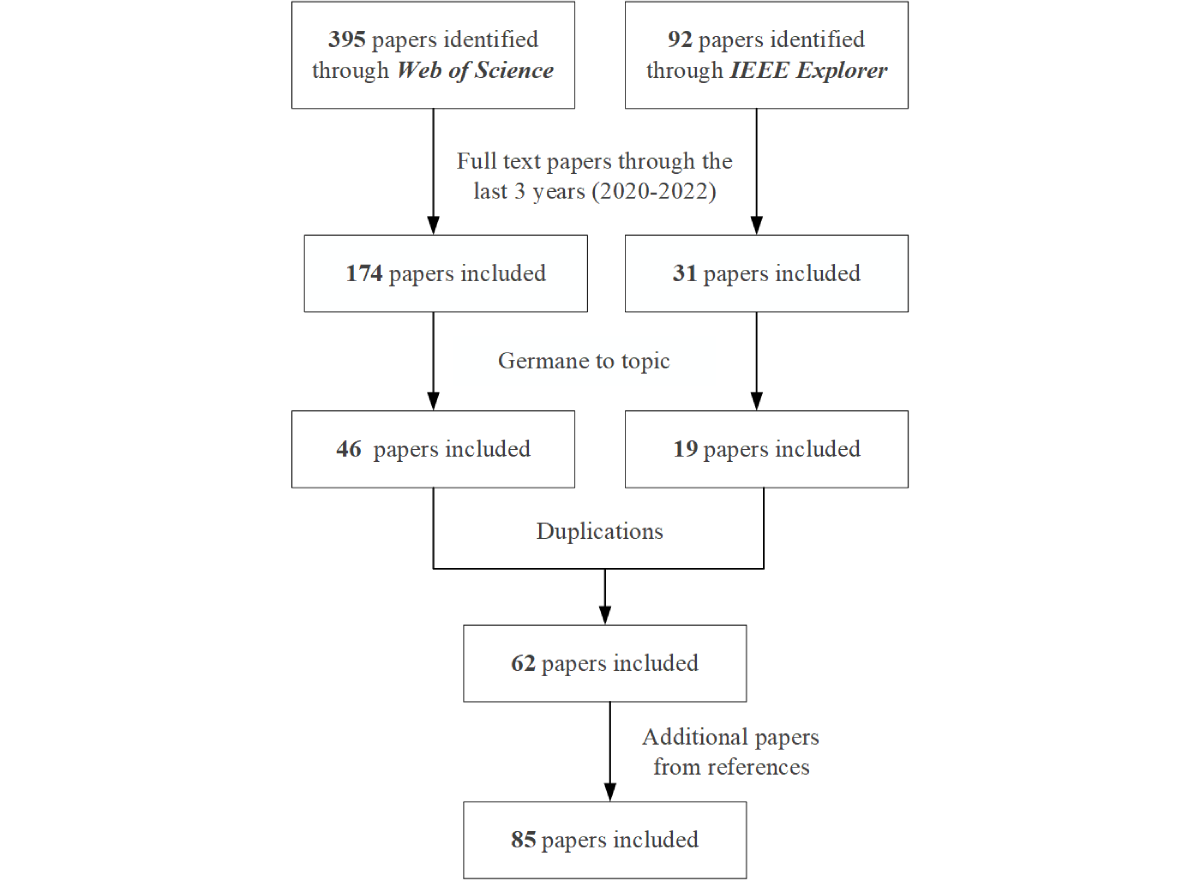
Flowchart of paper selection.

Figure 2 presents the paper selection flowchart used in this review. Initially, we identified a total of 487 papers in the Web of Science and IEEE databases by using the search term “FHIR” or “Fast Healthcare Interoperability Resources.” The time range of publications was set from January 1, 2020, to July 1, 2022, and we finalized 205 articles. After excluding those that merely mentioned the term FHIR but did not elaborate on it, 65 articles were retained. A check of duplications from this batch removed a further 3 articles. Finally, from the references of the remaining 62 articles, we found an additional 23 relevant articles, ending up with a total of 85 articles as the research materials of this study.

### Analysis Process

By carefully analyzing and collating the recent studies on the design of FHIR-based interoperable health care services, we derived the details of the FHIR PG.

We selected 85 FHIR-related articles and found that building FHIR-based health care services contains typically 3 stages, that is, data standardization, data management, and data integration. Each stage may use different practice methods, depending on the targeted scenarios and types of services.

The way to categorize these 85 articles is as follows: if an article’s main innovation feature focused on 1 of the 3 stages, we assigned it to the corresponding group. Specifically, we assigned those articles emphasizing the design process of FHIR profiles or proposing methods for migrating data from specific clinical data models (CDMs) to FHIR to the data standardization group, articles discussing the management of RESTful APIs to the data management group, and articles presenting approaches for integrating data with specific apps or platforms to the data integration group.

After categorizing the articles, we reviewed the key techniques used by each group to build their respective health care services. We compiled a general FHIR PG through this review. The workflow of the FHIR PG was derived by linking the stages, each consisting of multiple steps. It is important to note that alternative solutions might be identified for certain steps in the workflow based on different conditions. In addition, we leveraged the collective experience of our team working on health care IT projects to further refine and optimize the FHIR PG.

Finally, as an example, we arbitrarily selected a use case outside the scope of the reviewed articles and mapped it back to the FHIR PG to demonstrate the effectiveness and generalizability of the PG.

## Results

### Article Classification

#### Data Standardization

Data standardization typically involves two main steps: (1) defining profiles based on the data exchange requirements of interoperable services and (2) filling these profiles with the corresponding exchange data.

The base FHIR specification provides foundational resources applicable to various health care contexts. However, health care services often exhibit significant variability across different jurisdictions. Therefore, the base FHIR specification typically requires further adaptation, known as profile definition, to suit specific application contexts. Profile definition mainly encompasses three aspects: (1) rules about which resource elements to use and what additional elements to add to the base specification, (2) rules about which terminologies to use in particular elements, and (3) the restricted value range and cardinality of the elements.

Table 1 lists the typical profile definitions and the corresponding FHIR foundational resources discussed in the reviewed articles. As shown, these articles cover a wide range of categories, including genomics [10-14], imaging [15-17], cancer [18-20], diabetes [21,22], COVID-19 [23,24], infections [25], electrocardiography [26], screening [27], and allergy [28].

There are typically 2 approaches to filling the profiles with exchange data. One is redesigning the database to align with the FHIR resource structure, and the other is mapping data from an existing CDM-based legacy system to the FHIR-based system. Table 2 lists relevant articles discussing the latter approach. These articles could roughly fall into 7 groups based on the types of source CDMs. The groups include informatics for integrating biology and the bedside [29,30], Observational Medical Outcomes Partnership (OMOP) [31,32], OpenEHR [33,34], HL7 version 2 [35], variant call format [36], free text or arbitrary proprietary data [37,38], and multisource [39-42]. Multisource refers to cases where multiple CDMs are involved. For example, the study by Lenert et al [40] focused on transforming data from the OMOP and Patient-Centered Outcomes Research Network to FHIR. The study by Pfaff et al [39] aimed to transform data from informatics for integrating biology and the bedside, OMOP, and Patient-Centered Outcomes Research Network to FHIR. The study by Prud’hommeaux et al [41] compared 3 methods for transforming data from various source CDMs into FHIR. The study by Kiourtis et al [42] proposed a resource description framework transformation toolkit to combine FHIR and non-FHIR data.

The studies in Table 2 indicate that the transformation from a specific CDM type to FHIR typically involves a 2-step mapping process: model mapping and element mapping. Model mapping establishes a relationship between the original data model and the FHIR resource. Element mapping comprises 2 parts, key mapping and value mapping, which define how to map the data fields from the source CDM to the corresponding fields in the FHIR resources. The mapping rules observe the consensus-mapping relationships established by domain experts. These experts analyzed the semantic and structural differences between the source CDMs and FHIR and determined the appropriate mappings to ensure accurate and meaningful data transformation. Although current data transformation approaches intend to support specific source data and target FHIR resource types, it is worth noting that ongoing research and advancements in domain-based applied artificial intelligence, including natural language processing and deep learning, hold great potential for developing more generalized data transformation algorithms.

As highlighted in previous studies, the granularity of data plays a crucial role in data standardization. When the granularity of the source data is finer than that of the target data, there is potential for information loss during the transformation process: the severity of information loss increases with the extent of the granularity gap.

**Table 1:** Profile definitions from the reviewed articles.

Theme and study, year			Involved Fast Healthcare Interoperability Resources
**Genomics**			
	Murugan et al [10], 2021	DiagnosticReport, Specimen, ServiceRequest, Observation, and Task	
	Seong et al [11], 2021	MolecularSequence	
	Alterovitz et al [12], 2020	DiagnosticReport, ServiceRequest, and Observation	
	Klopfenstein et al [13], 2021	Questionnaire and Document	
	Khalifa et al [14], 2021	Patient, PractitionerRole, Organization, Specimen, ServiceRequest, Media, RiskAssessment, Task, MedicationRequest, CarePlan, DeviceRequest, NutritionOrder, SupplyRequest, and RequestGroup	
**Imaging**			
	Kohli et al [15], 2018	Patient, DiagnosticReport, ImagingStudy, AllergyIntolerance, Condition, MedicationOrder, Specimen, Organization, Practitioner, and Medication	
	Madrigal and Le [16], 2021	Media	
	Boufahja et al [17], 2021	Observation	
**Cancer**			
	Zong et al [18], 2021	Observation and DiagnosticReport	
	Gonzalez-Castro et al [19], 2021	Observation, Device, FamilyMemberHistory, AllergyIntolerance, Condition, Patient, MedicationStatement, Encounter, Questionnaire, QuestionnaireResponse, and Procedure	
	Zong et al [20], 2020	QuestionnaireResponse	
**Diabetes**			
	Ludmann et al [21], 2020	Observation	
	Glachs et al [22], 2020	Procedure, ProcedureRequest, Communication, Appointment, Observation, Condition, CommunicationRequest, Device, Encounter, Composition, Goal, Order, OrderResponse, MedicationAdministration, MedicationOrder, Organization, Patient, Practitioner, RiskAssessment, QuestionnaireResponse, Basic, and Parameters	
**COVID-19**			
	Bauer et al [23], 2021	Questionnaire	
	Sass et al [24], 2020	Procedure, Observation, Condition, DiagnosticReport, Procedure, Consent, Immunization, MedicationStatement	
**Infections**			
	Shivers et al [25], 2021	Consent, Coverage, DeviceUseStatement, Encounter, HealthcareService, Medication, MedicationAdministration, MedicationStatement, Observation, Patient, Practitioner, Procedure, ServiceRequest, and Specimen	
**Electrocardiogram**			
	Benhamida et al [26], 2020	Observation	
**Neonatal screening**			
	Bathelt et al [27], 2020	Patient, ServiceRequest, DiagnosticReport, Contract, Organization, and Practitioner	
**Allergy**			
	Lenivtceva and Kopanitsa [28], 2021	AllergyIntolerance	

**Table 2:** Data migration from the existing clinical data model to Fast Healthcare Interoperability Resources.

Study, year	Clinical data model of the source
Boussadi and Zapletal [29], 2017; Wagholikar et al [30], 2017	Informatics for integrating biology and the bedside
Jiang et al [31], 2017; Fischer et al [32], 2020	Observational Medical Outcomes Partnership
Ladas et al [33], 2022; Fette et al [34], 2020	OpenEHR
Xiao et al [35], 2021	HL7^a^ version 2
Dolin et al [36], 2021	Variant call format
Peterson et al [37], 2020; Wang et al [38], 2020	Free text or arbitrary proprietary
Lenert et al [40], 2021; Pfaff et al [39], 2019; Prud’hommeaux et al [41], 2021; Kiourtis et al [42], 2020	Multisource

^a^HL7: Health Level 7.

#### Data Management

Data management includes data storage and data exposure. Although FHIR defines 5 approaches for data exposure, including RESTful API, messaging, documents, services, and persistent store, recent articles predominantly chose to expose data in the form of APIs because of the rapid growth of the APIs economy. There are typically 2 methods for data management: developing a customized FHIR warehouse to store and manage FHIR data or selecting a mature third-party warehouse to handle the task.

Table 3 shows various data management choices and their corresponding targets. It reveals that developing a customized FHIR warehouse to maintain FHIR data often requires meeting some special service requirements. For instance, the customized FHIR warehouse developed by Demurjian et al [43] aimed to enable sensitivity and multilevel security controls. The one developed by Chatterjee et al [44] and Saripalle et al [45] served to integrate with specific terminology. The one developed by Ruminski et al [46], Saripalle [47], and Yu et al [48] intended to support multiple Internet of Things protocols. Finally, the one discussed in the studies by Khvastova et al [49], Dridi et al [50], Lee et al [51], Tanaka and Yamamoto [52], Cheng et al [53], Semenov et al [54], and Gruendner et al [55] was used to support data preprocess plug-ins.

On the other hand, several mature third-party platforms are available for managing FHIR data. In 2018, a total of 6 technology giants, including Amazon, Microsoft, Google, IBM, Oracle, and Salesforce, jointly announced that they would be committed to removing the barriers to adopting health care interoperability technologies, particularly those enabled through the cloud [56]. All these companies have launched FHIR data management platforms, providing FHIR data APIs for resource operations. Users of these platforms can store their data as FHIR resources and use the data APIs offered by the cloud platform for service development. For instance, the studies by Shi et al [57], Zampognaro et al [58], Ploner and Prokosch [59], and Kamel and Nagy [60] chose cloud warehouses, and the study by Mandl et al [61] chose an on-premises warehouse to rapidly deploy an FHIR development environment.

The abovementioned analysis highlights that choosing between proprietary and third-party warehouses involves trade-off considerations. Maintaining FHIR data through a proprietary warehouse offers 2 advantages: better privacy and greater flexibility for functional expansion. However, developing a proprietary warehouse requires extensive knowledge of FHIR standards and software development skills, resulting in higher costs. On the other hand, relying on third-party platforms offers the advantages of lower cost and higher implementation efficiency. However, storing sensitive data in a third-party warehouse, with the service provider not being the data owner, raises security and privacy concerns.

**Table 3:** Fast Healthcare Interoperability Resources (FHIR) data management methods and their corresponding targets.

Method and study, year		Target
**Develop FHIR warehouse**		
	Demurjian et al [43], 2020	Support lattice-based access control
	Chatterjee et al [44], 2022; Saripalle et al [45], 2020	Integrate with specific terminologies
	Ruminski et al [46], 2016; Saripalle [47], 2019; Yu et al [48], 2021	Support multiple IoT^a^ protocols
	Khvastova et al [49], 2020; Dridi et al [50], 2020; Lee et al [51], 2020; Tanaka and Yamamoto [52], 2020; Cheng et al [53], 2021; Semenov et al [54], 2019; Gruendner et al [55], 2021	Support data preprocess plug-ins
**Use third-party FHIR warehouse**		
	Shi et al [57], 2021; Zampognaro et al [58], 2021 Ploner and Prokosch [59], 2020; Kamel and Nagy [60], 2018	Rapidly deploy a development environment through a cloud FHIR warehouse
	Mandl et al [61], 2020	Rapidly deploy a development environment through an on-premises FHIR warehouse

^a^IoT: Internet of Things.

#### Data Integration

Data integration plays a vital role in health care across various domains, including service delivery, public health management, and clinical medicine or health care economics research, enabling better decision-making and improving overall health care outcomes. In service delivery, data integration is crucial for coordinating multiple IT systems, including the hospital information system (HIS), laboratory information system, picture archiving and communication system, EMR, and EHR. In public health, local governments need to collect health-related data within their jurisdictions to monitor regional health status and effectively address public health issues. In clinical medicine or health care economics research, it is essential to obtain data from diverse domains to conduct comprehensive studies and analyses.

There are 2 typical modes of FHIR data integration, as listed in Table 4.

The first mode of data integration is using an integrated service platform (ISPf). The ISPf is an orchestrating platform offering a series of API management functions such as API registration, API calling authorization, and API routing forward. Organizations wishing to exchange data through the ISPf must register their APIs on the platform. Other organizations can search for the appropriate APIs on the ISPf and make API calls. The ISPf performs API calling authorization to verify the calling rights and then routes the API calls to the respective organization to which the API belongs. This process facilitates data exchange among multiple organizations [62-75]. An example of this mode is the efficient transfer of medical records when a patient referral occurs.

The second mode of data integration is by way of interoperable apps. Different architectures can be selected for different application scenarios. In the case of apps with specific functions, such as statistics and analysis, SMART on FHIR would be a more efficient option [76-85]. In the case of apps with customized functions, such as supporting microservice architecture or blockchain architecture, customized architecture apps would be a more suitable option [86-94].

**Table 4:** Fast Healthcare Interoperability Resources data integration modes and their corresponding application scenarios.

Interoperable modes and study, year		Applied scenarios
**Integrated service platform**		
	Nan et al [62], 2021; Taechoyotin et al [63], 2021; Maxi and Morocho [64], 2022; Rosenau et al [65], 2022; Corici et al [66], 2020; Papaioannou et al [67], 2021; Hidayat and Hermanto [68], 2020; Sloane et al [69], 2021; Mukhiya and Lamo [70], 2021; Gruendner et al [71], 2022; Gruendner et al [72], 2020; Park et al [73], 2022; Ziminski et al [74], 2021; De et al [75], 2021	Control exchange data through APIs^a^ for service coordination among multiple organizations.
**App**		
	Suraj et al [76], 2022; Michaels et al [77], 2021; Curran et al [78], 2020; Thayer et al [79], 2021; Karhade et al [80], 2021; Wesley et al [81], 2021; Burkhardt et al [82], 2021; Hoffman et al [83], 2017; Stoldt and Weber [84], 2020; Stoldt and Weber [85], 2021	Substitutable Medical Applications and Reusable Technologies app: apps with specific functions, such as statistics and analysis.
	Alamri et al [86], 2021; George and Chacko [87], 2022; Gulden et al [88], 2021; Chaves et al [89], 2021; Bae and Yi [90], 2022; Bettoni et al [91], 2021; Weber et al [92], 2020; Sfat et al [93], 2021; Mohammed et al [94], 2021	Other architecture app: apps with customized functions, such as supporting microservice and blockchain architecture.

^a^API: application programming interface.

### FHIR PG Design

#### Practice Design

**Figure 3:**
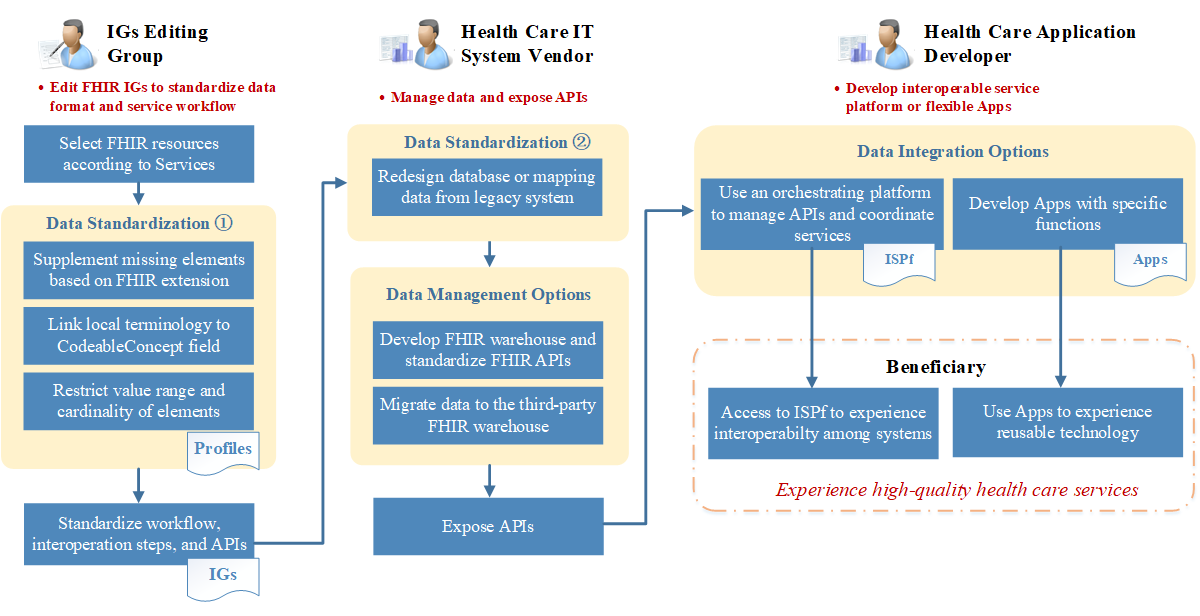
The general Fast Healthcare Interoperability Resources (FHIR) practice guideline—practice design. API: application programming interface; IG: Implementation Guide; ISPf: integrated service platform.

We present an FHIR practice design in Figure 3, which defines the responsibilities of stakeholders and outlines the complete practice process from data to services.

##### IGs Editing Group

The first stakeholder involved in the process is the IGs editing group, usually coordinated by a government agency or an institution with significant influence in the ecosystem. The primary responsibility of this group is to define the data and service models and release the IGs. The detailed processes are as follows. First, select necessary FHIR resources based on the service requirements. Second, for specific requirements beyond the scope of the original FHIR resources, the group needs to customize resource structure by FHIR profile. Profile generally involves 3 aspects: extending the data field by FHIR extension, linking the local CodeSystem to the CodeableConcept field of FHIR resources, and restricting the cardinality and ValueSet of FHIR foundational resource. The customized resources created by the profile enable better alignment with the data requirements in various scenarios. After completing the data unification task, the IGs editing group moves on to the unification of services workflow, which involves specifying the implementation steps in the workflow and standardizing the corresponding APIs. Ultimately, the abovementioned data and workflow specifications are integrated to form the comprehensive FHIR IGs that health care IT system vendors can adopt.

##### Health Care IT System Vendor

The second type of stakeholder is the health care IT system vendor, responsible for developing and maintaining systems, such as the HIS, laboratory information system, and picture archiving and communication system. First, the vendor must implement the IGs published by the IGs editing group, which involves standardizing data by redesigning the database according to the FHIR resource structure or mapping data from existing CDM-based legacy systems to FHIR-based systems. Second, with RESTful APIs, the vendor has 2 options for data exposure: either maintaining the FHIR data and APIs themselves or selecting a mature third-party platform. FHIR APIs must be exposed to support resource-level operations regardless of the chosen option.

It is worth pointing out that in terms of data exposure, FHIR defines 5 different approaches, and each data exposure approach has a different data integration method; it would be a lengthy discussion if all approaches are considered. To make FHIR PG more compatible with current technology stacks, we chose to focus on RESTful API rather than on other approaches in this study.

##### Health Care Application Developer

The third stakeholder involved in this process is the health care application developer, responsible for developing interoperable services using open FHIR APIs. As described in the *Data Integration* section, there are 2 typical modes. The first is to develop an ISPf, that is, an orchestrating platform, for service interoperability. The ISPf manages open APIs registered by each organization and enforces access specifications such as IGs, profiles, and workflows. Any IT systems accessing the ISPf and exchanging data must comply with these specifications. When an IT system needs to access multiple ISPfs, it must support multiple specifications. In such cases, the IT system can deploy an adapter above its native database to comply with various specifications. When the IT system acts as a producer, it reads the corresponding specifications from the adapter to expose the data. When it acts as a consumer, it reads the corresponding specifications from the adapter to parse data. The second mode is to develop specific apps that cater to specific requirements. For example, an app built with SMART on FHIR architecture supports a flexible and switchable application ecosystem.

##### Beneficiary

Beneficiaries such as hospitals, patients, public health institutions, and research institutions can benefit from high-quality FHIR-based health care services. For instance, if there is a need to exchange data through APIs to facilitate service coordination among multiple organizations, they can easily access the ISPf to fulfill this objective. Alternatively, they can choose a suitable app from the application gallery that caters to their needs and functions.

#### The Development Architecture for the Practice Design

##### Overview

**Figure 4:**
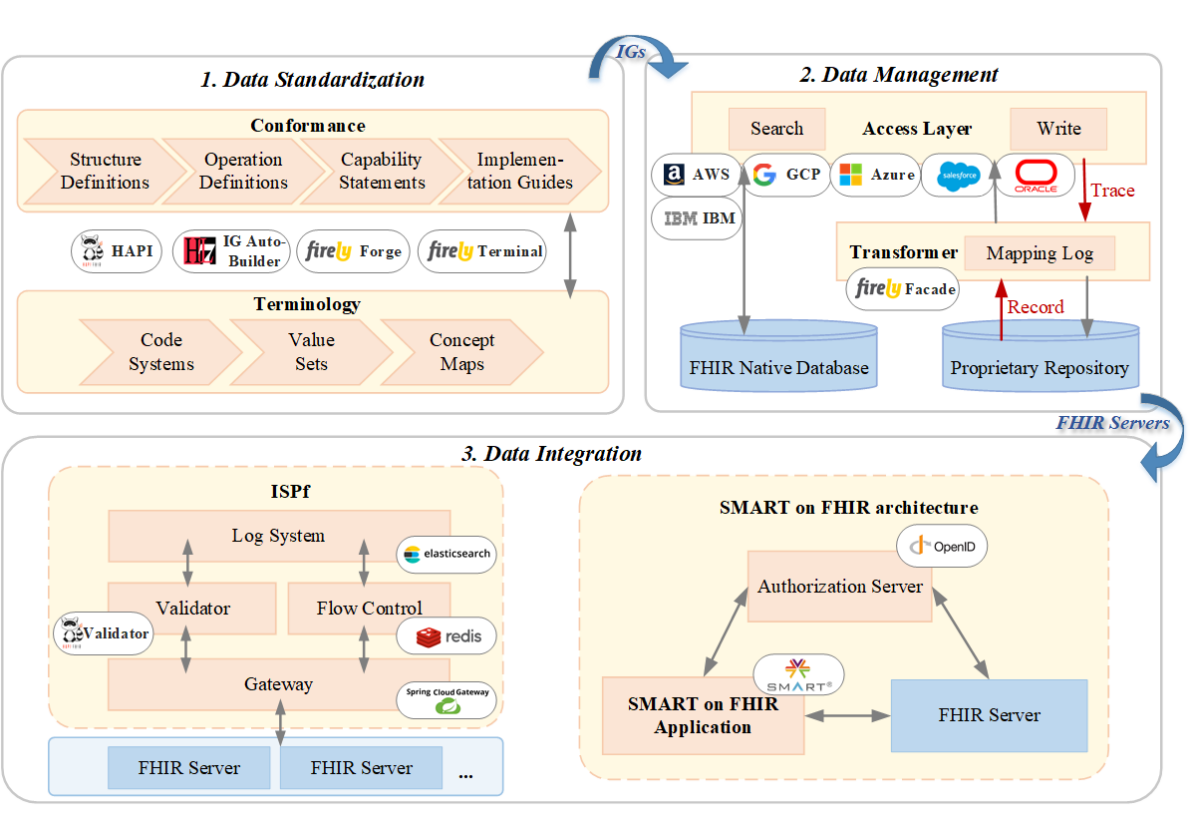
The general Fast Healthcare Interoperability Resources (FHIR) practice guideline—the development architecture for the practice design. IG: Implementation Guide; ISPf: integrated service platform; SMART: Substitutable Medical Applications Reusable Technologies.

We presented a 3-stage development architecture for the practice design, as shown in Figure 4. In addition, we compiled a list of commonly used tools in Table 5 to support the development process.

**Table 5:** A list of commonly used tools.

Tool and description					Availability
**Data standardization**					
		HAPI^a^ FHIR^b^	This tool provides Java API^c^ for HL7^d^ FHIR clients and servers	[95]	
		IG^e^ Auto-Builder	An IG publishing tool that makes your IGs to be visible on the internet [96]	[97]	
		Firely Forge	The official FHIR tool for managing FHIR profiles	[98]	
		Firely Terminal	A cross-platform command line tool with a range of commands for working with FHIR resources and installing and publishing FHIR packages	[99]	
**Data management**					
	Firely Facade		A special type of plug-in that registers services to access the existing data repository. It speaks FHIR in the front-end and talks directly to native data in the back-end	[100]	
	FHIR Works on AWS^f^		A framework used to deploy an FHIR server on AWS	[101]	
	FHIR server for Azure		An open-source implementation of FHIR specification designed for the Microsoft cloud	[102]	
	GCP^g^ Healthcare API		A cloud application that accelerates health care solution development with fully managed, enterprise-scale HL7 FHIR, HL7 version 2, and DICOM^h^ APIs	[103]	
	IBM FHIR server		An open-source Java solution that supports the processing, validation, and storage of health care data according to the HL7 FHIR specification	[104]	
	Oracle Healthcare Data Repository		The foundation of a health care information exchange platform that makes health care data more useful by supporting the integration and operation of a full spectrum of health care applications	[105]	
	Health Cloud		A tool that combines clinical and nonclinical customer data to drive efficiencies in health	[106]	
**Data integration**					
	Spring Cloud Gateway		This tool provides an API Gateway built on top of the Spring Ecosystem	[107]	
	Redis		This tool provides access to mutable data structures via a set of commands sent using a server-client model with TCP^i^ sockets and a simple protocol	[108]	
	Validator		The HAPI FHIR Validator API is a simple REST^j^ API to validate the structure and content of an FHIR object	[109]	
	Elasticsearch		A distributed, RESTful search and analytics engine is at the heart of the Elastic Stack	[110]	
	OpenID		An open standard and decentralized authentication protocol promoted by the nonprofit OpenID Foundation	[111]	
	OAuth		An open protocol to allow secure authorization in a simple and standard method from web, mobile, and desktop applications	[112]	
	SMART^k^		Define a workflow that an application can use to securely request access to data and then receive and use that data	[113]	

^a^HAPI: Health Level 7 application programming interface.

^b^FHIR: Fast Healthcare Interoperability Resources.

^c^API: application programming interface.

^d^HL7: Health Level 7.

^e^IG: Implementation Guide.

^f^AWS: Amazon Web Services.

^g^GCP: Google Cloud Platform.

^h^DICOM: Digital Imaging and Communications in Medicine.

^i^TCP: transmission control protocol.

^j^REST: representational state transfer.

^k^SMART: Substitutable Medical Applications Reusable Technologies.

##### Data Standardization

In the data standardization development stage, several components are defined to ensure the consistent use of codes within a specific context. The terminology system comprises essential resources such as CodeSystem, ValueSet, and ConceptMaps. These resources establish a framework for determining which codes can be used. Furthermore, the conformance system includes resources such as StructureDefinition, OperationDefinition, CapabilityStatement, and ImplementationGuide. These resources are crucial in creating profiles and IGs that adhere to a specific exchange framework. As mentioned in the *Data Standardization* section, the granularity of the data plays a crucial role in information loss. Pfaff et al [39] pointed out that information loss can be avoided by defining custom values or extensions during the data standardization stage. By incorporating custom values or extensions defined in this stage, it is possible to capture and preserve the finer-grained information that is likely to be lost during the transformation process.

During this process, developers can use various tools to facilitate efficient data standardization. The HL7 API (HAPI) FHIR offers a Java API for developing HL7 FHIR clients and servers. Forge serves as a management tool for FHIR profiles. The Firely Terminal, a cross-platform command line tool, provides a wide array of commands for working with FHIR resources and installing and publishing FHIR packages. IG Auto-Builder is another helpful tool that simplifies the creation and publication of IGs, available on the internet [96].

Ultimately, the data standardization stage would generate a set of IGs to ensure consistency and conformity in implementing higher-level services.

##### Data Management

Various situations can arise in the data management development stage, each bringing different challenges. These situations can fall into 3 options.

The first is to develop an FHIR-native warehouse that the health care IT system vendor manages. In this scenario, the vendor assumes responsibility for designing, implementing, and maintaining the warehouse.

The second is to select a well-established third-party warehouse, such as FHIR Works on Amazon Web Services, IBM FHIR Server, Google Cloud Platform Healthcare API, FHIR Server for Azure, Health Cloud, and Oracle Healthcare Data Repository, to store and explore the FHIR APIs. This approach allows vendors to leverage the capabilities of mature third-party warehouses for FHIR API functionality.

The third is to provide FHIR data using plug-ins. In this scenario, vendors retain their existing data infrastructure and use plug-ins to facilitate data transformation from its native format to the FHIR format. A tool called Facade is available to facilitate this mapping process.

As discussed in the *Data Standardization* section, the discrepancy in granularity between different systems can lead to potential information loss. To mitigate this issue, developers can incorporate a mapping log within the transformer component. When encountering a granularity gap during the mapping process, the mapping log captures and records the lost information, associating it with the corresponding target resource ID. This mapping log serves as a reference for any subsequent services or systems requiring detailed information about the mapping process. If the overlying services need to retrieve the lost information, they can make a request based on the resource ID recorded in the mapping log. This measure allows them to access the details lost during the initial mapping, ensuring that the required information is preserved and available for further analysis or processing.

Ultimately, the data management stage generates a series of FHIR APIs. These APIs serve as a foundation for data exploration and form the backbone of the infrastructure required for high-level services.

##### Data Integration

Two types of interoperable services are commonly used in the data integration development stage.

The first type is the ISPf, which enables interoperability among multiple organizations. The ISPf comprises 4 key components: gateway, validator, flow control, and log system. The gateway, built by the Spring Cloud Gateway, is responsible for API authorization and forwarding API requests between organizations. The validator ensures that the structure and content of the API data comply with the FHIR object defined in IGs. The HAPI FHIR Validator can build this functionality. The flow control component is designed to limit the number of simultaneous API calls to ensure a stable operation. Redis can effectively fulfill the flow control requirements. As ISPf manages multiple organizations and facilitates data exchange, maintaining a comprehensive log system is crucial for history tracking and auditing. Elasticsearch, a powerful search and analytics engine, can be used to develop the log system within the ISPf, enabling efficient storage and retrieval of API call records.

The second type of interoperable service is represented by apps built by the SMART on FHIR architecture [114]. This architecture consists of 3 key components: the resource server, authorization server, and the SMART on FHIR apps. The resource server is an access layer between the data management layer and the SMART on FHIR apps. The authorization server (an OpenID Connect–compliant web server) authenticates users and issues access tokens. SMART on FHIR apps is designed with specific functionalities and can be substituted based on user preferences.

#### Use Case

We arbitrarily selected a use case that was in addition to the reviewed articles. Portugal et al [115] designed a smart bed infrastructure with an HIS using FHIR. We mapped it back to the FHIR PG to demonstrate PG’s effectiveness and generalizability.

In this case, the roles and responsibilities can be mapped to the FHIR PG–practice design. The authors and their research partners formed an IGs editing group to define IGs consisting of profiles and workflows. The profiles were derived from foundational FHIR resources such as Observation, Device, and ServiceRequest. The workflows defined the frequency at which the smart bed would collect vital signs from the smart bed. Subsequently, the authors’ team, acting as a health care IT system vendor, developed a gateway that gathers raw data from sensors and converts it into FHIR for transmission. Although they did not discuss the final applications in detail, it can be inferred that health care application developers can build a better smart bed monitor based on their infrastructure.

The development architecture described in this paper can also be mapped back to the FHIR PG–development architecture. In the data standardization stage, the authors used the HAPI FHIR for HTTP processing, parsing and serialization, and FHIR REST semantics. It provided a bare-bones structure to build the API. In the data management stage, the authors developed a fog server as a gateway between the smart bed and HIS. This fog server is responsible for collecting raw data from the HIS, transforming it into the FHIR format, and facilitating its integration into the FHIR ecosystem. Finally, in the data integration stage, the authors enabled the HIS software to monitor patient procedures and flows, accompanied by the OAuth2 protocol for secure API communication.

## Discussion

### Principal Findings

FHIR has shown significant advantages in facilitating interoperability among health IT systems compared with established international standards. However, there are challenges in large-scale implementation and promotion, particularly in different countries. First, countries without incentive policies to encourage FHIR research and implementation may exhibit less enthusiasm for adopting FHIR standards. Second, the lack of a suitable infrastructure to support the implementation process can result in high costs associated with FHIR adoption. Third, the foundational resources provided by FHIR may not directly align with the specific service requirements in different regions, necessitating additional customization processes.

The following steps must be taken to address these challenges. First, it is crucial to have government policies that encourage the evolution and adoption of health care data standards. These policies can stimulate the enthusiasm and investment of stakeholders in the health care ecosystem to promote FHIR implementation on a larger scale. Second, strengthening the infrastructure helps reduce the cost and complexity associated with FHIR adoption, which includes developing services such as FHIR data storage, data standard quality control, and managed services for data operations. Third, FHIR profiles and workflows should be defined to address the specific requirements and characteristics of local health systems. By tailoring FHIR IGs to match the needs of different regions, the gap between FHIR foundational resources and specific service requirements can be bridged.

FHIR holds significant potential in standardizing health care data and promoting service interoperability among health care institutions. Its adoption can drive the transformation of the health care service model and enhance the overall quality of health care services. With the growing recognition of the benefits of FHIR and its demonstrated impact on health care interoperability, more stakeholders are expected to actively participate in enriching its implementation. This collective effort would lead to the emergence of extensive health care service innovations, further enhancing the delivery of high-quality health care services.

### Limitations

There are a few current limitations when applying the FHIR PG: (1) PG is derived from the waterfall model that follows a sequential and linear approach. Each step must be completed before proceeding to the next step. Therefore, it is time-consuming and costly to return and modify the previous steps if changes are necessary during the development process. (2) Although PG emphasizes the achievement of interoperability, it leaves out the security discussion. Developers must incorporate additional security mechanisms into PG–development architecture to ensure secure interoperation among multiple organizations.

### Conclusions

Owing to the unique characteristics of FHIR, including comprehensive coverage of data definitions, substantial flexibility of data exchange, explicit semantics, and many available open-source tools, FHIR-based services have attracted strong interest from stakeholders in the health care ecosystem. Current studies reveal that many institutions, such as hospitals, regulators, and researchers, have already begun collaborations in actively building FHIR foundational frameworks or application use cases. After conducting the latest literature review, we proposed a general FHIR PG to bridge the gap between FHIR IGs and the practice of building usable services. This PG helps stakeholders identify their participant roles, manage the scope of responsibilities, and develop relevant modules, which we believe would effectively facilitate the application and promotion of HL7 FHIR standards across the health care ecosystem.

## Abbreviations

API: application programming interface
CDA: Clinical Document Architecture
CDM: clinical data model
EHR: electronic health record
EMR: electronic medical record
FHIR: Fast Healthcare Interoperability Resources
HAPI: Health Level 7 application programming interface
HIS: hospital information system
HL7: Health Level 7
IG: Implementation Guide
ISPf: integrated service platform
OMOP: Observational Medical Outcomes Partnership
PG: practice guideline
SMART: Substitutable Medical Applications Reusable Technologies
